# Tetra-μ-acetato-κ^8^ *O*:*O*′-bis­[(*N*-ethyl­pyrimidin-2-amine)­copper(II)](*Cu*—*Cu*)

**DOI:** 10.1107/S1600536810034033

**Published:** 2010-08-28

**Authors:** Zaharah Aiyub, Edura Badaruddin, Zanariah Abdullah, Zainal A. Fairuz, Seik Weng Ng, Edward R. T. Tiekink

**Affiliations:** a Department of Chemistry, University of Malaya, 50603 Kuala Lumpur, Malaysia

## Abstract

In the centrosymmetric title mol­ecule, [Cu_2_(CH_3_COO)_4_(C_6_H_9_N_3_)_2_], each of the four acetate groups bridges a pair of Cu^II^ atoms [Cu—Cu = 2.6540 (4) Å]. The distorted octa­hedral geometry of the metal atom is completed by an *N*-donor atom of the *N*-ethyl­pyrimidin-2-amine ligand: an intra­molecular N—H⋯O hydrogen links its N—H group to an acetate carboxyl­ate O atom. In the crystal, C—H⋯O inter­actions link the mol­ecules into a supra­molecular chain along the *b* axis.

## Related literature

For related examples of tetra­kis­acetato­bis­[(substituted 2-amino­pyrid­yl)copper(II)] complexes, see: Fairuz *et al.* (2010*a* ▶,*b* ▶).
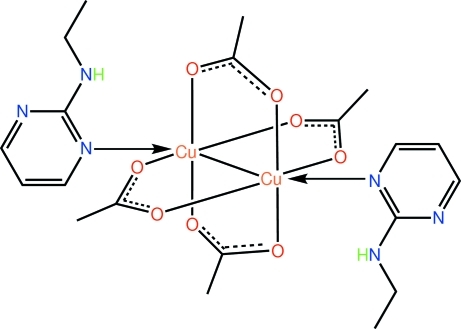


## Experimental

### 

#### Crystal data

[Cu_2_(C_2_H_3_O_2_)_4_(C_6_H_9_N_3_)_2_]
*M* *_r_* = 609.58
Triclinic, 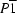

*a* = 7.8488 (6) Å
*b* = 8.5114 (7) Å
*c* = 10.2999 (8) Å
α = 98.404 (1)°
β = 92.698 (1)°
γ = 105.599 (1)°
*V* = 652.92 (9) Å^3^
*Z* = 1
Mo *K*α radiation
μ = 1.68 mm^−1^
*T* = 293 K
0.40 × 0.35 × 0.10 mm

#### Data collection

Bruker SMART APEX CCD diffractometer
Absorption correction: multi-scan (*SADABS*; Sheldrick, 1996 ▶) *T*_min_ = 0.613, *T*_max_ = 0.746
6208 measured reflections
2969 independent reflections
2669 reflections with *I* > 2σ(*I*)
*R*_int_ = 0.018

#### Refinement

*R*[*F* ^2^ > 2σ(*F* ^2^)] = 0.026
*wR*(*F* ^2^) = 0.080
*S* = 1.02
2969 reflections
170 parameters
1 restraint
H atoms treated by a mixture of independent and constrained refinement
Δρ_max_ = 0.28 e Å^−3^
Δρ_min_ = −0.25 e Å^−3^

### 

Data collection: *APEX2* (Bruker, 2009 ▶); cell refinement: *SAINT* (Bruker, 2009 ▶); data reduction: *SAINT*; program(s) used to solve structure: *SHELXS97* (Sheldrick, 2008 ▶); program(s) used to refine structure: *SHELXL97* (Sheldrick, 2008 ▶); molecular graphics: *ORTEP-3* (Farrugia, 1997 ▶) and *DIAMOND* (Brandenburg, 2006 ▶); software used to prepare material for publication: *publCIF* (Westrip, 2010 ▶).

## Supplementary Material

Crystal structure: contains datablocks global, I. DOI: 10.1107/S1600536810034033/hb5613sup1.cif

Structure factors: contains datablocks I. DOI: 10.1107/S1600536810034033/hb5613Isup2.hkl

Additional supplementary materials:  crystallographic information; 3D view; checkCIF report

## Figures and Tables

**Table 1:** Selected bond lengths (Å)

Cu—O1	1.978 (2)
Cu—O2^i^	1.963 (2)
Cu—O3	1.955 (1)
Cu—O4^i^	1.953 (1)
Cu—N1	2.246 (2)
Cu—Cu^i^	2.6540 (4)

Symmetry code: (i) 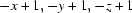.

**Table 2:** Hydrogen-bond geometry (Å, °)

*D*—H⋯*A*	*D*—H	H⋯*A*	*D*⋯*A*	*D*—H⋯*A*
N3—H3⋯O1	0.85 (1)	2.04 (1)	2.871 (2)	164 (2)
C4—H4a⋯O3^ii^	0.96	2.51	3.458 (3)	171

Symmetry code: (ii) 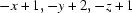.

**Fig. 1.**
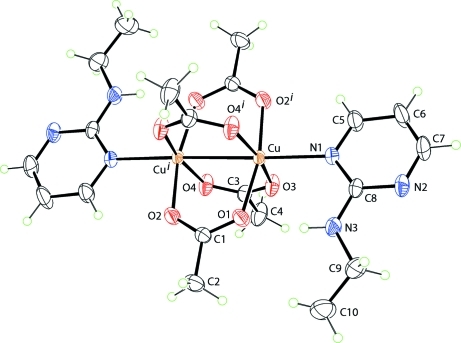
The molecular structure of (I) showing displacement ellipsoids at the 35% probability level. Primed atoms are related by the symmetry operation i: 1 - x, 1 - y, 1 - z.

**Fig. 2.**
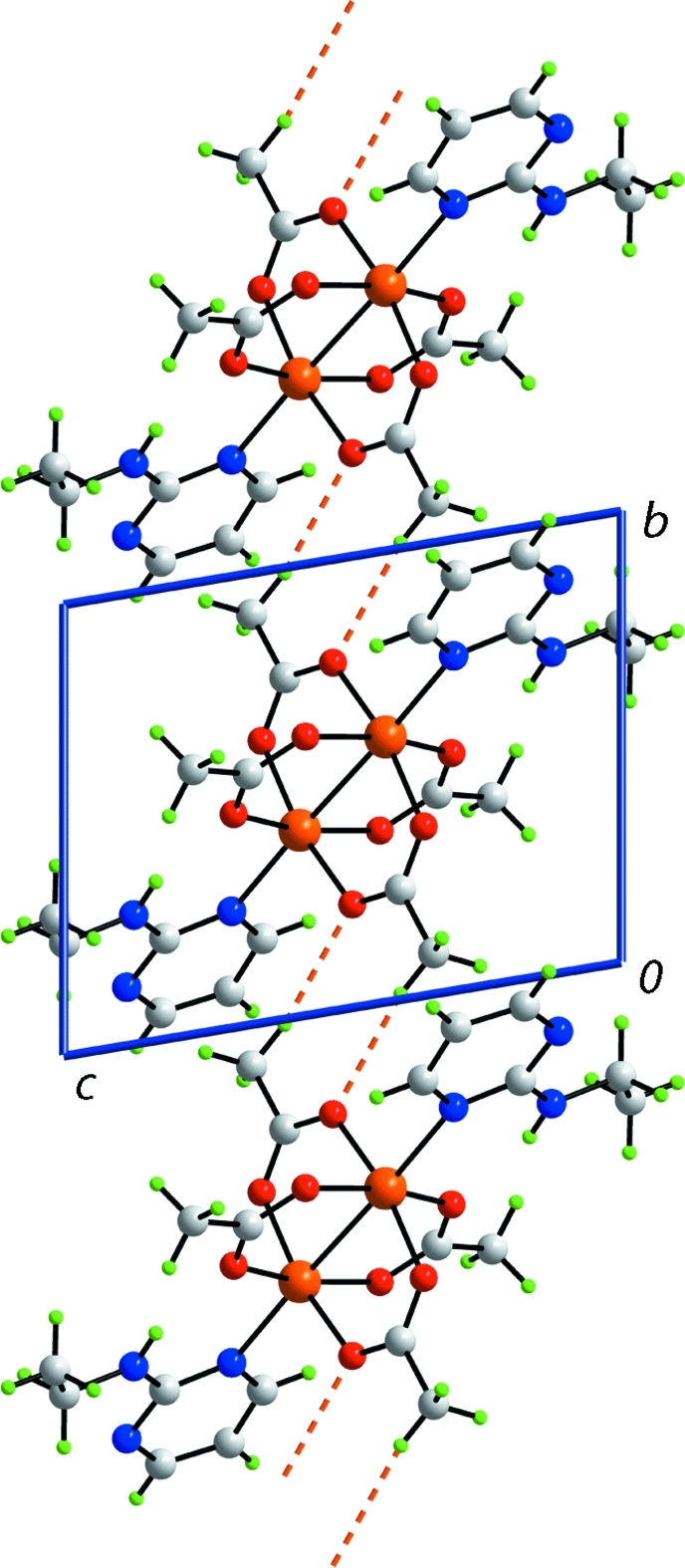
Supramolecular chain along the b axis in (I) mediated by C–H···O contacts shown as orange dashed lines.

## supplementary crystallographic information

### Comment

In continuation of on-going structural studies of tetrakisacetatobis[(substituted 2-aminopyridyl)copper] complexes (Fairuz *et al.*, 2010*a*, 2010*b*), the title complex, (I), was investigated.

The binuclear copper(II) complex, Fig. 1, is situated about a centre of inversion and features two Cu atoms bridged by four acetate groups. The Cu–O bond distances lie in a narrow range of 1.953 (1) to 1.978 (2) Å, Table 1. The coordination environment for each Cu atom is completed by an N atom derived from the *N*-ethylpyrimidin-2-amine ligand and the second Cu atom [Cu···Cu^i^ = 2.6540 (4) Å for *i*: 1 - *x*, 1 - *y*, 1 - *z*]. The resulting hexa-coordinated geometry is based on an octahedron. An intramolecular N3–H···O1 interaction contributes to the stability of the dinuclear molecule, Table 2. The *N*-heterocycle is effectively planar as seen in the C8–N3–C9–C10 torsion angle of -166.6 (2) °. In the crystal packing, the presence of C–H···O interactions connect dinuclear molecules into supramolecular chains along the *b* axis, Fig. 2 and Table 2.

### Experimental

Copper acetate (0.1 g, 0.5 mmol) was dissolved in acetonitrile (15 ml) and mixed with a solution of *N*-(pyrimidin-2-yl)ethylamine (0.2 g, 1.1 mmol) dissolved in acetonitrile (15 ml). The blue precipitate that formed was recrystallized from acetonitrile to yield blue prisms of (I).

### Refinement

Carbon-bound H-atoms were placed in calculated positions (C—H 0.93 to 0.97 Å) and were included in the refinement in the riding model approximation, with *U*_iso_(H) set to 1.2 to 1.5*U*_equiv_(C). The N-bound H-atom was located in a difference Fourier map and was refined with a distance restraint of N–H 0.86±0.01 Å; the *U_iso_* value was freely refined

### Crystal data

**Table:**

[Cu_2_(C_2_H_3_O_2_)_4_(C_6_H_9_N_3_)_2_]	*Z* = 1
*M**_r_* = 609.58	*F*(000) = 314
Triclinic, *P*1	*D*_x_ = 1.550 Mg m^−^^3^
Hall symbol: -P 1	Mo *K*α radiation, λ = 0.71073 Å
*a* = 7.8488 (6) Å	Cell parameters from 3508 reflections
*b* = 8.5114 (7) Å	θ = 2.5–28.2°
*c* = 10.2999 (8) Å	µ = 1.68 mm^−^^1^
α = 98.404 (1)°	*T* = 293 K
β = 92.698 (1)°	Prism, blue
γ = 105.599 (1)°	0.40 × 0.35 × 0.10 mm
*V* = 652.92 (9) Å^3^	

### Data collection

**Table:**

Bruker SMART APEX CCD diffractometer	2969 independent reflections
Radiation source: fine-focus sealed tube	2669 reflections with *I* > 2σ(*I*)
graphite	*R*_int_ = 0.018
ω scans	θ_max_ = 27.5°, θ_min_ = 2.0°
Absorption correction: multi-scan (*SADABS*; Sheldrick, 1996)	*h* = −10→10
*T*_min_ = 0.613, *T*_max_ = 0.746	*k* = −11→11
6208 measured reflections	*l* = −13→13

### Refinement

**Table:**

Refinement on *F*^2^	Primary atom site location: structure-invariant direct methods
Least-squares matrix: full	Secondary atom site location: difference Fourier map
*R*[*F*^2^ > 2σ(*F*^2^)] = 0.026	Hydrogen site location: inferred from neighbouring sites
*wR*(*F*^2^) = 0.080	H atoms treated by a mixture of independent and constrained refinement
*S* = 1.02	*w* = 1/[σ^2^(*F*_o_^2^) + (0.051*P*)^2^ + 0.1167*P*] where *P* = (*F*_o_^2^ + 2*F*_c_^2^)/3
2969 reflections	(Δ/σ)_max_ = 0.001
170 parameters	Δρ_max_ = 0.28 e Å^−^^3^
1 restraint	Δρ_min_ = −0.25 e Å^−^^3^

### Fractional atomic coordinates and isotropic or equivalent isotropic displacement parameters (Å^2^)

**Table:**

	*x*	*y*	*z*	*U*_iso_*/*U*_eq_	
Cu	0.60458 (3)	0.58733 (2)	0.42299 (2)	0.02818 (9)	
O1	0.3830 (2)	0.5272 (2)	0.30471 (15)	0.0479 (4)	
O2	0.2102 (2)	0.3821 (2)	0.43330 (16)	0.0491 (4)	
O3	0.5310 (2)	0.77124 (17)	0.51727 (16)	0.0474 (4)	
O4	0.3551 (2)	0.62484 (18)	0.64537 (16)	0.0473 (4)	
N1	0.7898 (2)	0.7417 (2)	0.30101 (17)	0.0328 (3)	
N2	0.8689 (3)	0.8709 (2)	0.11294 (19)	0.0473 (4)	
N3	0.5862 (3)	0.7135 (2)	0.12539 (18)	0.0435 (4)	
H3	0.513 (3)	0.648 (2)	0.165 (2)	0.048 (7)*	
C1	0.2361 (3)	0.4432 (2)	0.3307 (2)	0.0364 (4)	
C2	0.0792 (3)	0.4133 (4)	0.2317 (3)	0.0585 (7)	
H2A	0.0917	0.5081	0.1889	0.088*	
H2B	−0.0277	0.3946	0.2758	0.088*	
H2C	0.0736	0.3180	0.1672	0.088*	
C3	0.4244 (3)	0.7560 (2)	0.6037 (2)	0.0361 (4)	
C4	0.3760 (4)	0.9086 (3)	0.6633 (3)	0.0595 (7)	
H4A	0.3991	0.9880	0.6044	0.089*	
H4B	0.4457	0.9552	0.7459	0.089*	
H4C	0.2522	0.8799	0.6775	0.089*	
C5	0.9529 (3)	0.8135 (3)	0.3561 (2)	0.0497 (6)	
H5	0.9836	0.7926	0.4389	0.060*	
C6	1.0798 (3)	0.9180 (4)	0.2965 (3)	0.0650 (8)	
H6	1.1928	0.9706	0.3381	0.078*	
C7	1.0308 (3)	0.9397 (3)	0.1740 (3)	0.0566 (6)	
H7	1.1151	1.0064	0.1302	0.068*	
C8	0.7529 (3)	0.7767 (2)	0.18107 (19)	0.0342 (4)	
C9	0.5238 (4)	0.7487 (3)	0.0013 (2)	0.0564 (6)	
H9A	0.5716	0.6922	−0.0707	0.068*	
H9B	0.5645	0.8666	0.0000	0.068*	
C10	0.3254 (4)	0.6923 (4)	−0.0163 (3)	0.0754 (9)	
H10A	0.2848	0.7136	−0.0996	0.113*	
H10B	0.2785	0.7513	0.0534	0.113*	
H10C	0.2855	0.5759	−0.0140	0.113*	

### Atomic displacement parameters (Å^2^)

**Table:**

	*U*^11^	*U*^22^	*U*^33^	*U*^12^	*U*^13^	*U*^23^
Cu	0.02719 (13)	0.02865 (14)	0.02816 (14)	0.00393 (9)	0.00634 (9)	0.00911 (9)
O1	0.0309 (7)	0.0684 (10)	0.0392 (8)	−0.0011 (7)	−0.0011 (6)	0.0225 (7)
O2	0.0326 (7)	0.0646 (10)	0.0454 (9)	−0.0021 (7)	−0.0016 (6)	0.0253 (8)
O3	0.0602 (10)	0.0330 (7)	0.0548 (10)	0.0145 (7)	0.0295 (8)	0.0149 (7)
O4	0.0601 (10)	0.0336 (7)	0.0526 (9)	0.0139 (7)	0.0292 (8)	0.0122 (7)
N1	0.0319 (8)	0.0309 (8)	0.0339 (8)	0.0039 (6)	0.0071 (6)	0.0077 (6)
N2	0.0493 (11)	0.0492 (10)	0.0452 (10)	0.0069 (8)	0.0209 (9)	0.0206 (9)
N3	0.0444 (10)	0.0495 (10)	0.0344 (9)	0.0033 (8)	0.0042 (8)	0.0178 (8)
C1	0.0308 (9)	0.0396 (10)	0.0367 (10)	0.0061 (8)	−0.0001 (8)	0.0081 (8)
C2	0.0364 (11)	0.0801 (18)	0.0541 (15)	0.0041 (11)	−0.0089 (10)	0.0223 (13)
C3	0.0414 (10)	0.0314 (9)	0.0367 (10)	0.0108 (8)	0.0080 (8)	0.0068 (8)
C4	0.0823 (18)	0.0381 (12)	0.0678 (17)	0.0253 (12)	0.0358 (14)	0.0144 (11)
C5	0.0410 (12)	0.0578 (14)	0.0434 (12)	−0.0030 (10)	0.0060 (10)	0.0171 (11)
C6	0.0387 (12)	0.0742 (18)	0.0664 (18)	−0.0164 (12)	0.0095 (12)	0.0203 (14)
C7	0.0510 (14)	0.0589 (15)	0.0581 (15)	0.0006 (11)	0.0247 (12)	0.0254 (12)
C8	0.0419 (11)	0.0298 (9)	0.0325 (10)	0.0094 (8)	0.0137 (8)	0.0080 (7)
C9	0.0632 (16)	0.0740 (17)	0.0362 (12)	0.0191 (13)	0.0054 (11)	0.0222 (11)
C10	0.0622 (17)	0.116 (3)	0.0530 (16)	0.0251 (17)	−0.0021 (13)	0.0323 (17)

### Geometric parameters (Å, °)

**Table:**

Cu—O1	1.978 (2)	C2—H2A	0.9600
Cu—O2^i^	1.963 (2)	C2—H2B	0.9600
Cu—O3	1.955 (1)	C2—H2C	0.9600
Cu—O4^i^	1.953 (1)	C3—C4	1.504 (3)
Cu—N1	2.246 (2)	C4—H4A	0.9600
Cu—Cu^i^	2.6540 (4)	C4—H4B	0.9600
O1—C1	1.247 (2)	C4—H4C	0.9600
O2—C1	1.246 (3)	C5—C6	1.378 (3)
O3—C3	1.247 (2)	C5—H5	0.9300
O4—C3	1.250 (2)	C6—C7	1.355 (4)
N1—C5	1.321 (3)	C6—H6	0.9300
N1—C8	1.349 (3)	C7—H7	0.9300
N2—C7	1.331 (3)	C9—C10	1.494 (4)
N2—C8	1.340 (3)	C9—H9A	0.9700
N3—C8	1.338 (3)	C9—H9B	0.9700
N3—C9	1.448 (3)	C10—H10A	0.9600
N3—H3	0.85 (1)	C10—H10B	0.9600
C1—C2	1.502 (3)	C10—H10C	0.9600
			
O4^i^—Cu—O3	167.23 (6)	H2B—C2—H2C	109.5
O4^i^—Cu—O2^i^	88.86 (8)	O3—C3—O4	125.51 (18)
O3—Cu—O2^i^	89.53 (8)	O3—C3—C4	117.20 (18)
O4^i^—Cu—O1	89.76 (8)	O4—C3—C4	117.29 (18)
O3—Cu—O1	88.95 (7)	C3—C4—H4A	109.5
O2^i^—Cu—O1	166.94 (6)	C3—C4—H4B	109.5
O4^i^—Cu—N1	97.38 (6)	H4A—C4—H4B	109.5
O3—Cu—N1	95.36 (6)	C3—C4—H4C	109.5
O2^i^—Cu—N1	93.54 (6)	H4A—C4—H4C	109.5
O1—Cu—N1	99.52 (6)	H4B—C4—H4C	109.5
O4^i^—Cu—Cu^i^	83.74 (4)	N1—C5—C6	123.0 (2)
O3—Cu—Cu^i^	83.49 (4)	N1—C5—H5	118.5
O2^i^—Cu—Cu^i^	84.07 (5)	C6—C5—H5	118.5
O1—Cu—Cu^i^	82.87 (5)	C7—C6—C5	116.3 (2)
N1—Cu—Cu^i^	177.35 (4)	C7—C6—H6	121.9
C1—O1—Cu	124.63 (14)	C5—C6—H6	121.9
C1—O2—Cu^i^	123.95 (14)	N2—C7—C6	123.7 (2)
C3—O3—Cu	123.73 (13)	N2—C7—H7	118.2
C3—O4—Cu^i^	123.48 (13)	C6—C7—H7	118.2
C5—N1—C8	115.85 (17)	N3—C8—N2	116.97 (19)
C5—N1—Cu	115.99 (14)	N3—C8—N1	117.50 (17)
C8—N1—Cu	128.08 (13)	N2—C8—N1	125.53 (19)
C7—N2—C8	115.5 (2)	N3—C9—C10	109.8 (2)
C8—N3—C9	123.88 (19)	N3—C9—H9A	109.7
C8—N3—H3	118.0 (17)	C10—C9—H9A	109.7
C9—N3—H3	118.1 (17)	N3—C9—H9B	109.7
O2—C1—O1	124.4 (2)	C10—C9—H9B	109.7
O2—C1—C2	117.62 (19)	H9A—C9—H9B	108.2
O1—C1—C2	117.9 (2)	C9—C10—H10A	109.5
C1—C2—H2A	109.5	C9—C10—H10B	109.5
C1—C2—H2B	109.5	H10A—C10—H10B	109.5
H2A—C2—H2B	109.5	C9—C10—H10C	109.5
C1—C2—H2C	109.5	H10A—C10—H10C	109.5
H2A—C2—H2C	109.5	H10B—C10—H10C	109.5
			
O4^i^—Cu—O1—C1	84.62 (19)	Cu^i^—O2—C1—C2	−177.97 (16)
O3—Cu—O1—C1	−82.68 (19)	Cu—O1—C1—O2	−2.0 (3)
O2^i^—Cu—O1—C1	0.7 (4)	Cu—O1—C1—C2	178.01 (16)
N1—Cu—O1—C1	−177.93 (18)	Cu—O3—C3—O4	2.6 (3)
Cu^i^—Cu—O1—C1	0.90 (18)	Cu—O3—C3—C4	−177.68 (17)
O4^i^—Cu—O3—C3	−2.8 (4)	Cu^i^—O4—C3—O3	−2.2 (3)
O2^i^—Cu—O3—C3	−85.53 (19)	Cu^i^—O4—C3—C4	178.11 (17)
O1—Cu—O3—C3	81.49 (18)	C8—N1—C5—C6	−0.1 (3)
N1—Cu—O3—C3	−179.05 (18)	Cu—N1—C5—C6	−177.0 (2)
Cu^i^—Cu—O3—C3	−1.45 (17)	N1—C5—C6—C7	−2.4 (4)
O4^i^—Cu—N1—C5	−93.37 (16)	C8—N2—C7—C6	0.3 (4)
O3—Cu—N1—C5	85.80 (16)	C5—C6—C7—N2	2.3 (4)
O2^i^—Cu—N1—C5	−4.07 (16)	C9—N3—C8—N2	−3.6 (3)
O1—Cu—N1—C5	175.63 (16)	C9—N3—C8—N1	175.8 (2)
Cu^i^—Cu—N1—C5	21.6 (10)	C7—N2—C8—N3	176.3 (2)
O4^i^—Cu—N1—C8	90.11 (16)	C7—N2—C8—N1	−3.1 (3)
O3—Cu—N1—C8	−90.72 (16)	C5—N1—C8—N3	−176.44 (19)
O2^i^—Cu—N1—C8	179.42 (16)	Cu—N1—C8—N3	0.1 (3)
O1—Cu—N1—C8	−0.89 (17)	C5—N1—C8—N2	3.0 (3)
Cu^i^—Cu—N1—C8	−154.9 (8)	Cu—N1—C8—N2	179.51 (15)
Cu^i^—O2—C1—O1	2.0 (3)	C8—N3—C9—C10	−166.6 (2)

Symmetry codes: (i) −*x*+1, −*y*+1, −*z*+1.

### Hydrogen-bond geometry (Å, °)

**Table:**

*D*—H···*A*	*D*—H	H···*A*	*D*···*A*	*D*—H···*A*
N3—H3···O1	0.85 (1)	2.04 (1)	2.871 (2)	164 (2)
C4—H4a···O3^ii^	0.96	2.51	3.458 (3)	171

Symmetry codes: (ii) −*x*+1, −*y*+2, −*z*+1.
